# House dust mites atopy patch test - an important screening tool for detection of house dust mites allergy?

**DOI:** 10.1186/2045-7022-5-S1-O19

**Published:** 2015-03-11

**Authors:** Barbara Jasiewicz-Honkisz, Ida Marchewka, Krystyna Targosz

**Affiliations:** 1 Department of Internal Diseases and Rural Medicine, Jagiellonian University, Cracow; Poland; 2 Jagiellonian University, Cracow, Department of Internal Diseases and Rural Medicine, Cracow; Poland; 3 Centrum Medyczne Med-ALL, Poradnia Alergologiczna, Kraków, Poland

## Background

The frequency of contact allergens is often regionally different and hence it regional identification is important for the prevention of allergic contact dermatitis (ACD).

## Objectives

Aim of this study was evaluation of the frequency of particular positive results of patch test (APT).

## Methods

A total number of 127 patients from Outpatient Allergic Clinic MED-ALL Medical Centre (age 2-76 years old) with suspected ACD were examined using the most common allergens from the patch tests from the European Standard Series including nickel, pallatium, chrome, cobalt, fragrance-mix I and II, balsam of Peru, formaldehyde and propolis and one extra allergen -- house dust mite (HDM). The data obtained were subjected to statistical analysis.

## Results

The highest positivity of APT was seen against HDM (67%), then nickel (51%), chrome (40%), cobalt (39%), pallatium (16%), propolis (16%), fragrance-mix I (13,3%), balsam of Peru (11,6%), fragrance-mix II (9,8%) formaldehyde (4,1%). In the group of patients with positive results with HDM higher percentage is in younger group<18 years old (70%) with almost 68% of cases with strong positive results (++ or +++).

## Conclusion

HDM allergens play an important role in determining the clinical severity of allergic dermatitis and should be included in basic patch test series.

